# When can physical distancing be relaxed? A health production function approach for COVID-19 control policy

**DOI:** 10.1186/s12889-021-11088-x

**Published:** 2021-06-02

**Authors:** Dradjad H. Wibowo

**Affiliations:** ABFI Institute Perbanas, Jakarta Selatan, DKI, Jakarta Indonesia

**Keywords:** COVID-19, Physical distancing, Pandemic control policy, State of transmission, Health production function, Production elasticity, Developing countries

## Abstract

**Background:**

To assess if physical distancing measures to control the COVID-19 pandemic can be relaxed, one of the key indicators used is the reproduction number *R*. Many developing countries, however, have limited capacities to estimate *R* accurately. This study aims to demonstrate how health production function can be used to assess the state of COVID-19 transmission and to determine a risk-based relaxation policy.

**Methods:**

The author employs a simple “bridge” between epidemiological models and production economics to establish the cumulative number of COVID-19 cases as a short-run total product function and to derive the corresponding marginal product, average product, and production elasticity. Three crucial dates defining the states of transmission, labelled red, yellow, and green zones, are determined. Relaxation policy is illogical in the “red zone” and is not recommended in the “yellow zone”. In the “green zone”, relaxation can be considered. The Bayesian probability of near term’s daily cases meeting a policy target is computed. The method is applied to France, Germany, Italy, the UK, and the US, and to Indonesia as an example of application in developing countries.

**Results:**

This study uses data from the WHO COVID-19 Dashboard, beginning from the first recording date for each country until February 28, 2021. As of June 30, 2020, France, Germany, Italy, and the UK had arrived at the “green zone” but with a high risk of transmission re-escalations. In the following weeks, their production elasticities were rising, giving a signal of accelerated transmissions. The signal was corroborated by these countries’ rising cases, making them leaving the “green zone” in the later months. By February 28, 2021, the UK had returned to the “green zone”, France, Germany, and Italy were still in the “yellow zone”, while the US reached the “green zone” at a very high number of cases. Despite being in the “red zone”, Indonesia relaxed its distancing measures, causing a sharp rise of cases.

**Conclusions:**

Health production function can show the state of COVID-19 transmission. A rising production elasticity gives an early warning of transmission escalations. The elasticity is a useful parameter for risk-based relaxation policy.

**Supplementary Information:**

The online version contains supplementary material available at 10.1186/s12889-021-11088-x.

## Introduction

To control the rapid spread of the coronavirus disease 2019 (COVID-19) pandemic, many countries employ community-wide physical (social) distancing measures. These measures usually include border closing, movement restriction, school, workplace, and public-place closures, prohibition of gatherings, isolation or quarantine. These measures constitute a pandemic control policy termed “large-scale public health restrictions” by the World Health Organization (WHO) [1]

The short-term economic costs of physical distancing, however, can be very high. At the macro level, a 6-weeks social distancing is estimated to lower the Gross Domestic Product (GDP) of 15 European countries by 4.3–9.2% [2]. Using an Effective Lockdown Index (ELI), a 14% contraction of the global GDP on a year-on-year basis has also been estimated [3]. At the micro level, physical distancing adversely affects household income. It is estimated that the US’ household income could decline by 4.6–25.6% [4].

At the global level, in June 2020 the International Monetary Fund (IMF) estimates that the COVID-19 pandemic and its containment policies could subdue the 2020 global economic growth to − 4.9%, an 8.2% correction from its January 2020 projection [5]. In its June 2020 Economic Outlook, the Organisation for Economic Co-operation and Development (OECD) projects a 6% contraction of the world’s economy in a single-hit scenario, and a 7.6% fall if there is a second wave of infections before the end of 2020 [6]. This contraction will cause higher global unemployment.

Notwithstanding the short-term economic costs, in the long-term the economic benefits of social distancing have been shown to outweigh the costs. Over a 30-year planning horizon using a 3% discount rate, effective social distancing produces economic net benefits of about US$ 5.2 trillion [7]. Evidence from the 1918 Flu Pandemic in the US also shows the economic benefits of social distancing as a non-pharmaceutical intervention (NPI). Cities that intervened earlier and more aggressively with an NPI had an increased economy after the pandemic [8].

If social distancing is not applied, the economic costs can be much higher. A two-sector analysis of the US Input-Output Table shows that without social distancing, the falls in output, capacity utilization and investment is around two-folds of those with social distancing [9]. If social distancing goes wrong, the economy could experience another severe hit [9]. If social distancing is “just slightly too relaxed”, the net economic result would be worse than doing nothing [10].

Once distancing measures are applied, given their high economic costs, the policy challenge facing governments is to determine when the measures can be relaxed without increasing the risk of transmission re-escalations. In this case, WHO recommends that COVID-19 transmission should come under control [1], based on a number of epidemic indicators, most notably the reproduction number *R*. Developed countries such as Germany use *R* as a benchmark to ease lockdown; its *R* prior to the easing was 0.8 [11]. The UK frequently releases its *R* and growth rate figures, which as of June 25, 2020 were 0.7–0.9 and − 4% to − 2%, respectively [12]. The UK government did not, however, solely rely on these indicators to ease lockdown measures.

For developing countries, estimating R can be very problematic. Many developing countries have very limited financial and research capacities to estimate R accurately on a daily basis. They have a relatively inferior health data collection system, making them unable to accurately estimate the basic reproduction number R_0_ in the early stages of a pandemic. Fiscal tightness limits their ability to conduct large-scale test and tracing programs. With only a tiny fraction of the population is tested, the accuracy of any estimate of R is highly questionable.

Given this lack of reliable R estimates in developing countries, the author aims to demonstrate how short-run health production function can be used as an additional approach to assess the state of COVID-19 transmission and to determine a risk-based pandemic control policy. The author employs a simple “bridge” between epidemiological models and production economics to treat cumulative number of COVID-19 cases as a short-run total product function. The methods are very simple that developing country officials can perform them easily.

## Methods

Since Grossman’s seminal work [13], there have been a large body of research on health production function. These researches use community or individual health status as the output variable, measured for example by morbidity [14], mortality or individual health status, with inputs such as health care, safe water and sanitation, habits (e.g. diet, smoking) and other relevant variables.

In this study, the author constructs a health production function from the infected compartment *I(t)* in the Susceptible-Infected-Recovered (SIR) model and determines the state of transmission using the steps below. These steps are formally presented in Additional file 1.

- **Step 1.** Let *I(t)* be the number of COVID-19 cases at *t*, where *t* = time. Because the number of cases is reported daily, the day is used as the unit of time. For *t* = 0, 1, 2, …, *τ* days, we have *Y* (*τ*) *= Y* (*τ* − 1) *+ I* (*τ*), where *Y* (*τ*) = cumulative number of COVID-19 cases on the *τ-*th day, *Y* (*τ* − 1) *=* cumulative number of COVID-19 cases on the *(τ* − 1*)-*th day, and *I* (*τ*) = daily number of COVID-19 cases on the *τ-*th day. It follows that *I*(*t*) = *dY*(*t*)/*dt*. Because *I*(*t*) ≠ *c* where *c =* constant, the integral of *dY*(*t*) = *I*(*t*) *dt* is at least twice-differentiable with respect to (w.r.t) *t.* Note that for *t* = 0, *Y(0) = I(0) = 0,* and for *t* = 1, *Y(1) = I(1).*
- **Step 2.** Take the cumulative number of COVID-19 cases (*Y(t)*) as the health status output. The input variables might include daily population mobility, physical distancing measures, daily weather, holiday season, daily vaccination, and other relevant variables. Physical distancing measures might be treated as dummy variables representing their application or non-application. The UK’s four tier rules, for example, can be treated this way.

The study, however, is not aimed at analysing the relative contribution of each input on *Y(t)*. Thus for simplicity purpose, all inputs are collapsed into a single variable, i.e. the time variable. Consequently, we have a short-run health production function. The main departure of *Y(t)* from the standard function in economics is that it has no downward curve. This is because *I(t)* does not take a negative value, and hence, *Y(t)* does not decrease unless there is a significant change in case definition or an incorrect recording of data.

**Fig. 1:**
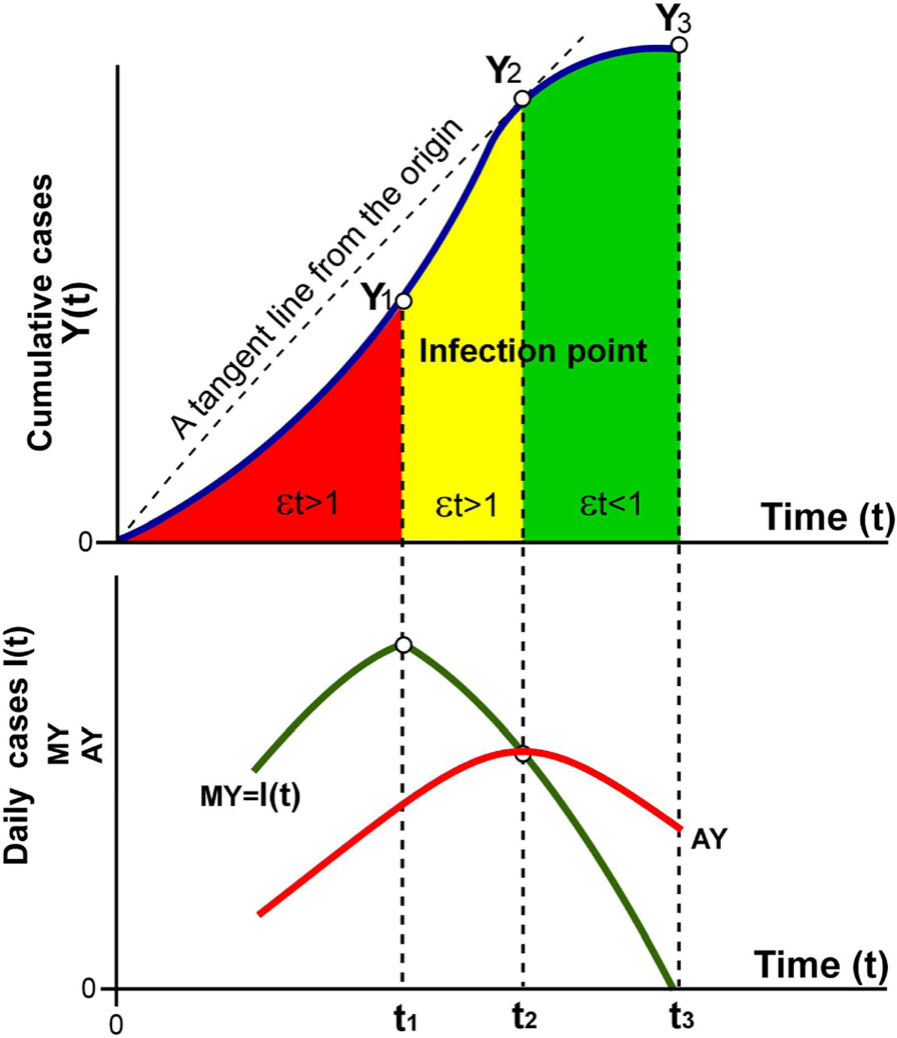
The relationship between Y(t), MY=I(t), and AY

- **Step 3.** Smooth out *Y(t)‘s* time-series data to have clearer trends. Assuming an incubation period of 5 days as employed by Kucharski et al. [15], the author uses 5-day exponential moving average (EMA). The technique has another benefit. If on a given day *I(t)* shows a negative entry, the averaging process from the next 5 days smooth out its effects, provided that the negative is not too large.
- **Step 4.** Derive from *Y(t)* the corresponding marginal product of the infected (*MY*), the average product of the infected (*AY*), and the production elasticity of the infected w.r.t time *t* (*ℇ t*). Note that *ℇ t* is defined as the percentage change in *Y(t)* for every 1 % change in *t*. Because *dt* = one reporting day, one can calculate *MY*, *AY*, and *ℇ t* from cumulative number of COVID-19 data straightforwardly, without any need to first find the functional form of *Y(t)*. This study uses the terms *MY* and *I(t)* interchangeably because *MY* = *I(t)*. For reason of definition rigor [16], this study uses arc elasticity, even though estimates of both arc- and point-elasticity are presented.
- **Step 5.** Determine the state of transmission and draw pandemic control inferences from *Y*(*t*), *MY*, *AY*, and *ℇ t* for *I(t)* ≥0 by use of Fig. 1 showing the canonical relationship among total product, marginal product, average product, and production elasticity. The inferences depend on whether three crucial dates determining the state of transmission, i.e. *t*_*1*_, *t*_*2*_, or *t*_*3*_, have been reached, as described below:

1. Before *t*_*1*_ is reached, *MY*=*I(t)* has not peaked and is still rising, and *ℇ t* > 1. Policy makers need to apply physical distancing measures to stop the rise, and to bring the number of daily cases down. This state is represented by the “red zone” in Fig. 1.
2. At *t*_*1*_, *MY* reaches its peak, which corresponds to the inflection point *Y*_*1*_. From *t*_*1*_ to *t*_*2*_, *I(t)* declines. Naturally, policy makers start to think if distancing measures can be relaxed. But at this state of transmission, *MY* > *AY* and *ℇ t* > 1. Relaxing the measures is not recommended. This state is represented by the “yellow zone” in Fig. 1.
3. At *t*_*2*_, *AY* reaches its peak and *ℇ t* = 1. From *t*_*2*_ to *t*_*3*_, both *MY* and *AY* decline, *MY* ≤ *AY*, and 0 ≤ *ℇ t* ≤ 1. Relaxation of distancing measures can be considered at this state of transmission, depicted by the “green zone” in Fig. 1.
4. At *t*_*3*,_ *MY*=*I(t)* = 0. No more daily COVID-19 cases are recorded; *Y(t)* reaches its steady-state.

At any time *t* in the “green zone”, the question is then “is now the right time to relax the measures?” To answer this, assuming that policy makers rationally adopt risk-based decision-making, the author assesses the Bayesian probability of near term’s daily COVID-19 cases being equal to or below a given daily-cases target of I*. This probability is conditional on *ℇ* t because 0 ≤ *ℇ t* ≤ 1 must be satisfied. To showcase inference #2, the probability is computed from *t*_*1*_. I* may be set in accordance to the number of daily-cases that a health system can handle, or be determined arbitrarily based on, say, a socio-political process. A rational policy maker will only relax distancing measures if the probability is high. A more cautious one might add another target such as “constant or declining number of daily-cases”.

**S**teps 1–2 outline a simple “bridge” between the SIR model and production economics. Steps 3–5 are performed in Microsoft Excel.

## Results

To test this approach, the author initially analyses COVID-19 cases in France, Germany, Italy, the UK and the US. The method is then applied to Indonesia as a developing country example, given the author’s familiarity with its health data system. The first manuscript was completed in July 2020, covering a period from the first day a confirmed case is recorded until June 30, 2020, termed as the initial period. In this manuscript, the period of analysis is extended to February 28, 2021, termed as the updated period. All data are obtained from the WHO [17].

### The initial period

Additional file 2 provides detailed results from the initial period. Some of the key findings are:

1. France, Germany, Italy, and the UK had reached *t*_*1*_ with Italy being the earliest one and the UK the latest one. The US and Indonesia had not, so they were still in the “red zone”.
2. France, Germany, Italy, and the UK had reached *t*_*2*_. Thus, they had arrived at the “green zone”. With regard to t_3_, none of the countries studied had reached the steady-state of *Y(t)*.
3. Among the European countries studied, the UK had the largest *ℇ t*, while Italy has the smallest.
4. If after reaching *t*_*1*_ a country still exhibited *ℇ t* > 1, the probability of *I(t + 1)* ≤ I* is zero. The probability is slightly above zero if a higher I* is set. These results support inference #2 outlined in Step 5 of the Methods section.
5. For 0 ≤ *ℇ t* ≤ 1, France, Germany, and Italy had the probabilities of 0.69, 0.41, and 0.53, respectively, to meet a policy target of I* = 500 daily-cases. At 0 ≤ *ℇ t* ≤ 0.3, the probabilities were 0.95, 0.94, and 0.79, respectively. With an additional target *I(t + 1)* ≤ *I(t)*, for 0 ≤ *ℇ t* ≤ 1, the probabilites were 0.38, 0.29, and 0.37, respectively.
6. For the UK, I* = 500 was unattainable. It had a probability of 0.34 and 0.82 to meet I* = 1000 at 0 ≤ *ℇ t* ≤ 1 and 0 ≤ *ℇ t* ≤ 0.5, respectively. With *I(t + 1)* ≤ *I(t)* policy target, at 0 ≤ *ℇ t* ≤ 0.5 the probability was 0.55. The 0 ≤ *ℇ t* ≤ 0.3 range was not applicable because the UK’s lowest elasticity for the April 25–June 30, 2020 period was 0.33.
7. France, Germany, Italy, and the UK had a relatively high risk of their daily-cases failing to meet the policy target or even rising.

### The updated period

#### The state of transmission

**Fig. 2:**
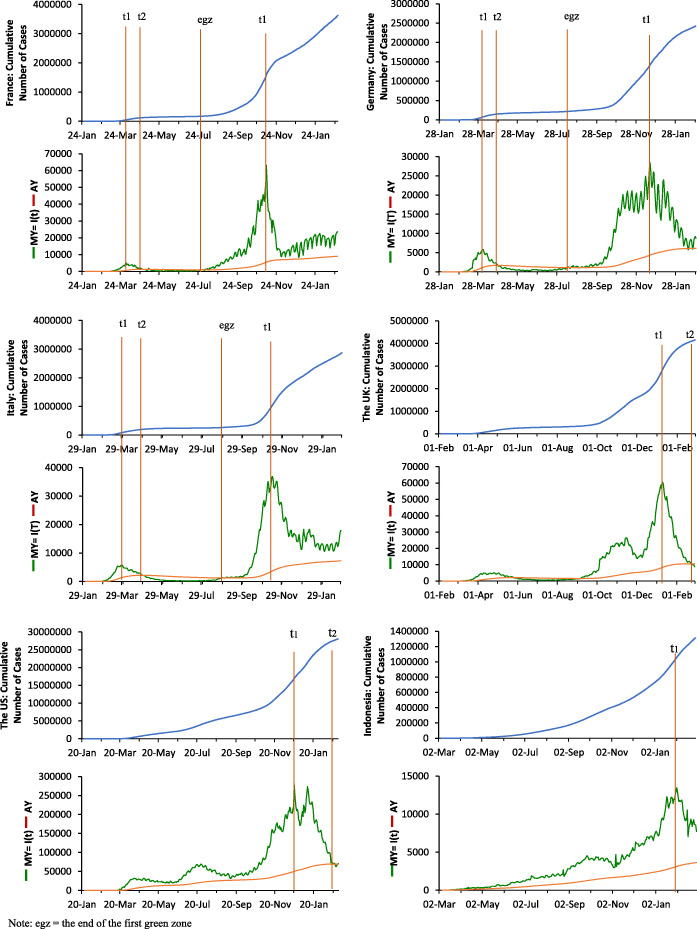
Cumulative number of cases, daily-cases, and average product of the infected (Until Feb 2021)

Table 1 presents descriptive statistics of the data. Figure 2 shows the 5-day EMA curves of *Y(t)*, *MY*=*I(t)*, and *AY.* Detailed data, calculations, and figures are available in Additional files 3, 4, 5, 6, 7, 8.

**Table 1:** Descriptive statistics

	France	Germany	Italy	The UK	The US	Indonesia
Recording dates	Jan 24, 2020-Feb 28, 2021	Jan 28, 2020-Feb 28, 2021	Jan 29, 2020-Feb 28, 2021	Feb 1, 2020-Feb 28, 2021	Jan 20, 2020- Feb 28, 2021	Mar 2, 2020- Feb 28, 2021
Number of recording days (*t*)	402	398	397	394	406	364
Cumulative number of cases, *Y(t)*, on Feb 28, 2021						
Orignal data	3,671,208	2,442,336	2,907,825	4,170,523	28,174,978	1,329,074
EMA	3,624,075	2,424,819	2,871,999	4,152,888	28,033,244	1,313,619
Number of daily-cases, *MY=I(t)*, 5-day EMA						
Mean	9106	6154	7308	10,648	69,734	3649
Standard deviation	11,251	7570	9378	13,893	69,084	3461
Coefficient of variation	124%	123%	128%	130%	99%	95%
Maximum value	63,259	28,383	36,818	60,530	280,412	13,475
Average product of the infected, *AY*, 5-day EMA						
Mean	3004	2124	2672	3304	25,894	1229
Standard deviation	3044	1850	2234	3158	21,307	1049
Coefficient of variation	101%	87%	84%	96%	82%	85%
Maximum value	Not applicable	Not applicable	Not applicable	10,606	69,423	Not applicable
Crucial dates (… - Jun 30, 2020)						
*t*_*1*_	Apr 1, 2020	Apr 5, 2020	Mar 29, 2020	Apr 25, 2020	Not Applicable	Not Applicable
*t2*	Apr 26–28, 2020	Apr 26–27, 2020	Apr 29–30, 2020	May 24–25, 2020	Not Applicable	Not Applicable
Crucial dates (…- Feb 28, 2021)						
End of the first green zone	Jul 28, 2020	Aug 13, 2020	Aug 29, 2020	Sep 5, 2020	Not Applicable	Not Applicable
*t1*	Nov 8, 2020	Dec 19,2020	Nov 15, 2020	Jan 9, 2021	Dec 20, 2020	Jan 31, 2021
*t2*	Not Applicable	Not Applicable	Not Applicable	Not Applicable	Not Applicable	Not Applicable

After June 30, 2020, *MY* in France, Germany, Italy and the UK rose and exceeded *AY* from July 28, August 13, August 29, and September 5, 2020, respectively. It means, these countries began to leave the “green zone”. France, Germany, and Italy regained *t*_*1*_ in November–December 2020, but failed to reach *t*_*2*_ until February 28, 2021. The UK regained *t*_*1*_ on January 9, 2021 and *t*_*2*_ on February 23, 2021, at an *MY* of 60,530 and an *AY* of 10,606, much worse than the initial period’s corresponding levels of 5007 and 2127, respectively. The US reached *t*_*1*_ on December 20, 2020 and *t*_*2*_ on February 22, 2021, at an extremely high *MY* of 280,412 and *AY* of 69,423, respectively. None of the countries studied had reached the steady-state of *Y(t)* at *t*_*3*_.

#### Production elasticities

Table 2 presents the arc production elasticities (*ℇ t*). Point elasticity values are also presented for comparative purpose. In general, arc elasticities are larger than point elasticities. Italy has the smallest *ℇ t*, with a mean of 2.94. This means that for every 1 % change in time, Italy has 2.94% additional COVID-19 cases. The US exhibits the largest *ℇ t* with a maximum value of 36.11.

**Table 2:** The elasticity of production

	France	Germany	Italy	The UK	The US	Indonesia
Arc elasticity of production, 5-day EMA						
Mean	3.45	3.08	2.94	3.34	3.60	2.90
Standard deviation	3.52	3.38	3.78	2.94	4.03	0.64
Coefficient of variation	102%	110%	128%	88%	112%	22%
Maximum value	18.51	18.41	31.01	13.75	36.11	5.16
Minimum value *)	0.04	0.01	0.00	0.05	0.10	0.44
Point elasticity of production, 5-day EMA						
Mean	3.27	2.92	2.76	3.18	3.38	2.80
Standard deviation	3.19	3.05	3.28	2.70	3.37	0.61
Coefficient of variation	98%	104%	119%	85%	100%	22%
Maximum value	14.71	15.49	19.40	11.69	27.59	4.51
Minimum value *)	0.04	0.01	0.0	0.05	0.10	0.46

Note: *) It excludes minimum values in the beginning of transmission

**Fig. 3:**
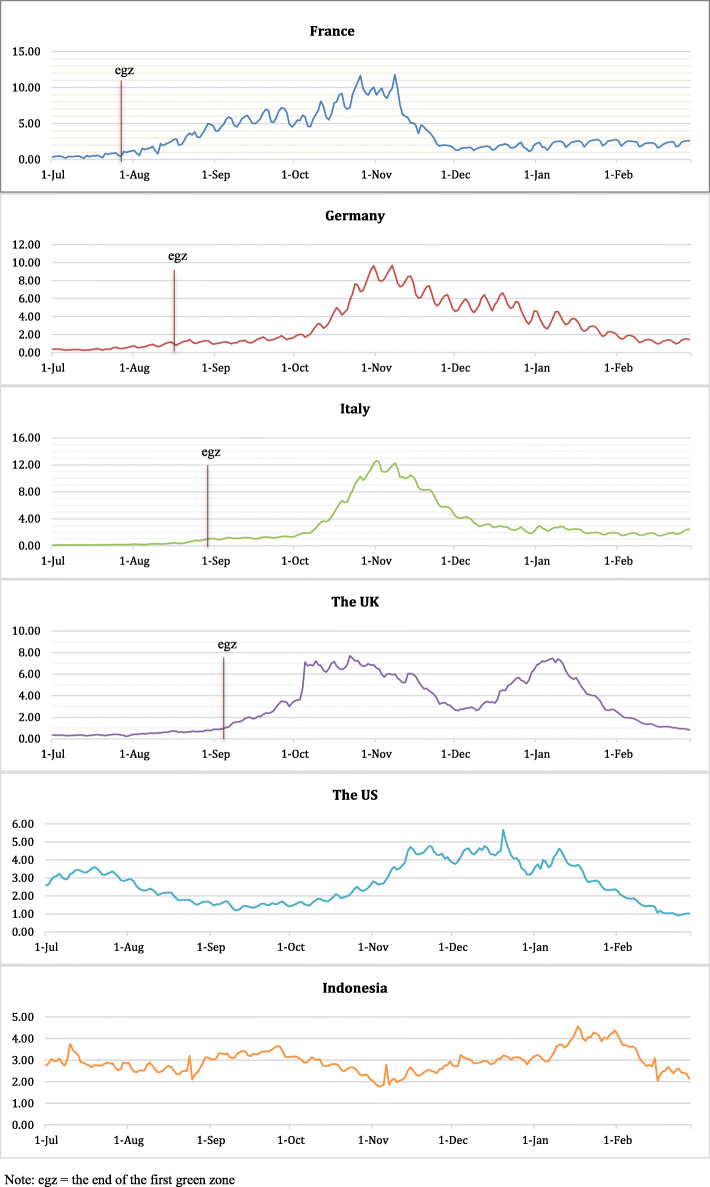
Arc elasticities (5-day EMA), July 1, 2020 – Feb 28, 2021

Figure 3 presents *ℇ t* curves for the countries studied. More complete figures are available in Additional file 9. The curves show that *ℇ t* was on the rise in the weeks leading up to the end of France’s, Germany’s, Italy’s and the UK’s first “green zone”. This result suggests that a rising *ℇ t* in the “green zone” can give early warning of case escalations.

#### Probability of a policy target

Because the UK and the US had arrived at the “green zone”, this analysis is applicable to them. However, I* = 1000 daily-cases was no longer attainable for the UK. It needed a much higher I* = 10,000. For the US, an I* = 65,000 is applied. Table 3 presents the probability of *I(t + 1)* ≤ I*, given a range of *ℇ t*.

**Table 3:** Probability of a policy target

	France	Germany	Italy	The UK	The US	Indonesia
Policy (daily-cases) target, I*	This analysis is not applicable for France	This analysis is not applicable for Germany	This analysis is not applicable for Italy	10,000	65,000	This analysis is not applicable for Indonesia
Probability of I (t + 1) ≤ I*, if:						
ɛt > 1.0				0.00	0.00	
0 ≤ ɛt ≤ 1.0				0.50	0.25	
0 ≤ ɛt ≤ 0.5				Not applicable	Not applicable	
Probability of I (t + 1) ≤ I* and I (t + 1) ≤ I(t), if:						
ɛt > 1.0				0.00	0.00	
0 ≤ ɛt ≤ 1.0				0.50	0.25	
0 ≤ ɛt ≤ 0.5				Not applicable	Not applicable	

For *ℇ t* > 1 after reaching *t*_*1*_, both the UK and the US showed a zero probability of meeting *I(t + 1)* ≤ I*. The probability was slightly above zero if a higher I* was set. For example, for I* = 11,500 the UK’s probability was 0.09. As do the initial period’s results, these results support inference #2 outlined in Step 5 of the Methods section.

For 0 ≤ *ℇ t* ≤ 1, the UK and the US had the probabilities of 0.50 and 0.25, respectively, to meet their policy target I*. If *I(t + 1)* ≤ *I(t)* was targeted, each of the UK and the US had only 4 cases satisfying 0 ≤ *ℇ t* ≤ 1 from their respective *t*_*1*_ until February 28, 2021. The probabilites returned the same values.

#### Application to a developing country: Indonesia

In January–February 2020 Indonesia denied that the country has a COVID-19 case. When the central government finally announced the “first” case on March 2, opportunity to estimate *R*_*0*_ more accurately had been wasted. Consequently, Indonesia has no reliable estimates of *R* until today.

Using this study’s methods, it was found that Indonesia had not reached *t*_*1*_ in the initial period (Additional file 2). Yet since June 1, 2020 Indonesia began to relax its “Large Scale Social Restrictions”. Restrictions such as school closure were still in place, but the country opened its shopping malls, allowed domestic holidays, and did not stop some crowd gatherings despite them being a criminal offence. Consequently, Indonesia’s daily-cases continued to rise until January 2021.

On January 31, 2021 Indonesia reached *t*_*1*_ at an *MY* of 13,475, shifting its state of transmission from the “red zone” to the “yellow zone”. Given Indonesia’s relaxed distancing measures, and the fact that its vaccination program just began on January 13, 2021, what caused the shift needs to be thoroughly analysed in future studies.

## Discussion

This study demonstrates how short-run health production function is employed to assess the state of COVID-19 transmission, using only data on the cumulative number of cases and the recording dates. The data are processed in relatively simple methods, which can be performed at minimal costs in developing countries.

This study also shows that relaxing physical distancing measures can only be considered when the state of transmission is in the “green zone”. In this zone the probability of maintaining a relatively low number of near term’s daily COVID-19 cases, at a given elasticity range, is relatively high. In the “yellow zone” the probability is zero or near zero.

As of June 30, 2020, France, Germany, Italy, and the UK had arrived at the “green zone”. But because their probabilities of meeting the “constant or declining number of daily-cases” target were below 0.5 at 0 ≤ *ℇ t* ≤ 1, they had a high risk of transmission re-escalations. This result was confirmed by these countries’ rising cases after June 30, 2020, causing them to leave the “green zone” in the later months.

As of February 28, 2021, France, Germany, Italy and Indonesia were still in the “yellow zone”. Some forms of distancing measures are still needed to bring down the transmission, in addition to vaccination program. The UK and the US had reached the “green zone”, but they still had a high risk of transmission re-escalations because of their low probability to meet the policy targets.

A higher production elasticity with respect to the time input means that for every 1 % change in time, we have a higher percentage of additional cases, or in other words, a faster transmission. Because this elasticity represents “transmission speed”, it explains why a rising production elasticity in the “green zone” can give early warning of transmission escalations.

Simplicity and minimum data requirement are key features of this study. More advanced statistics might be employed, but doing so requires more efforts from health officials. To show this, the author performs regression and curve-fitting exercises to the UK data using Eviews and Matlab, respectively, presented in Additional file 10.

## Conclusions

Short-run health production function can be used as an additional method to assess the state of transmission and to determine a risk-based physical distancing relaxation policy. Given its simplicity and minimum data requirement, the approach can be very useful for developing countries which for various reasons are unable to estimate *R*_*0*_ thoroughly and accurately.

### Follow-up research

With the “bridge” outlined in this study, many researches beneficial for public health and economic policy-making could be undertaken. Estimating a health production function with multiple inputs, including a distancing measure, is an obvious example. Such a study could help answer the question of how a given distancing measure affects the trajectory of an epidemic. The corresponding total cost function could also be derived, and a cost effective mix of public health restrictions could be determined. One could go further by exploring the link between analytical solutions to the SIR model, health production function parameters, and the labor supply and economic growth models. How infection and recovery rates in the SIR and other compartmental models affect economic growth or financial market indices could also be investigated. Application of this study’s methods to other epidemics is another possibility.

## Supplementary Information

**Additional file 1.** Cumulative number of COVID-19 cases as a short-run health production function; a formal mathematical presentation of the method used in this study.

**Additional file 2.** Figures; The initial period’s results.

**Additional file 3.** country name_rem1. Country name_COVID-19; All data collected and processed in this study, including the calculation formula of each cell.

**Additional file 4.** country name_rem1. Country name_COVID-19; All data collected and processed in this study, including the calculation formula of each cell.

**Additional file 5.** country name_rem1. Country name_COVID-19; All data collected and processed in this study, including the calculation formula of each cell.

**Additional file 6.** country name_rem1. Country name_COVID-19; All data collected and processed in this study, including the calculation formula of each cell.

**Additional file 7.** country name_rem1. Country name_COVID-19; All data collected and processed in this study, including the calculation formula of each cell.

**Additional file 8.** country name_rem1. Country name_COVID-19; All data collected and processed in this study, including the calculation formula of each cell.

**Additional file 9.** Arc elasticity based on 5-day EMA; Arc elasticity values and graphs.

**Additional file 10.** Eviews and matlab exercises; Eviews and matlab results for the UK.

## Data Availability

COVID-19 data used in this study, covering a period from the first recording day until February 28, 2021, are openly available at WHO [17]. Permission from WHO is not needed to use the data. All data generated or analysed during this study are included in this published article and its Additional files 3, 4, 5, 6, 7, 8, 9. These additional files have been deposited in a reusable format in the figshare repository under DOI: 10.6084/m9.figshare.13047575 [18].

## Abbreviations

COVID-19: Coronavirus disease 2019
CV: Coefficient of variation
ELI: Effective lockdown index
EMA: Exponential moving average
GDP: Gross Domestic Product
IMF: International Monetary Fund
NPI: Non-pharmaceutical intervention
OECD: Organisation for Economic Co-operation and Development
SIR: Susceptible-Infected-Recovered
WHO: World Health Organization.

## References

[1] WHO. Strengthening and adjusting public health measures throughout the COVID-19 transition phases. Policy considerations for the WHO European Region. WHO Regional Office for Europe, Copenhagen, 2020. https://www.euro.who.int/en/health-topics/health-emergencies/coronavirus-covid-19/technical-guidance/2020/strengthening-and-adjusting-public-health-measures-throughout-the-covid-19-transition-phases.-policy-considerations-for-the-who-european-region,-24-april-2020 [accessed 7 June 2020].

[2] Barrot J, Grassi B, Sauvagnat J. Sectoral effects of social distancing. Published online April 15, 2020. 10.2139/ssrn.3569446 [accessed 6 June 2020].

[3] Hatzius J, Tilton A, Struyven D. Global economics analyst measuring the impact of lockdowns and social distancing on global GDP. Published online April 26, 2020. https://www.gspublishing.com/content/research/en/reports/2020/04/27/3a0089c7-c1d1-4243-8dbd-da6141a501be.html [accessed 6 June 2020].

[4] Strong A, Welburn JW. An estimation of the economic costs of social-distancing policies. 2020. Santa Monica: RAND Corporation. https://www.rand.org/content/dam/rand/pubs/research_reports/RRA100/RRA173-1/RAND_RRA173-1.pdf [accessed 6 June 2020].

[5] IMF. World Economic Outlook Reports**-**World Economic Outlook Update, June 2020: A crisis like no other, an uncertain recovery IMF: Washington, 2020. https://www.imf.org/en/Publications/WEO/Issues/2020/06/24/WEOUpdateJune2020 [accessed 26 June 2020].

[6] OECD. OECD economic outlook, June 2020: the world economy on a tightrope. OECD, Paris, 2020. https://www.oecd.org/economic-outlook/june-2020/ [accessed 12 June 2020].

[7] Thunström L, Newbold SC, Finnoff D, Ashworth M, Shogren JF. The benefits and costs of using social distancing to flatten the curve for COVID-19**.** Forthcoming *J Benefit Cost Anal* 2020; published online April 14, 2020. 10.2139/ssrn.3561934 [accessed 6 June 2020].

[8] Correia S, Luck S, Verner E. Pandemics depress the economy, public health interventions do not: evidence from the 1918 flu. Published online March 30, 2020. 10.2139/ssrn.3561560 [accessed 6 June 2020].

[9] Bodenstein M, Corsetti G, Guerrieri L. Social distancing and supply disruptions in a pandemic. May 2, 2020. Cambridge-INET Working Paper Series No: 2020/17 Cambridge Working Papers in Economics: 2031. https://www.inet.econ.cam.ac.uk/working-paper-pdfs/wp2017.pdf [accessed 6 June 2020].

[10] Maharaj S, Kleczkowski A. Controlling epidemic spread by social distancing: do it well or not at all*.* BMC Public Health 2012; 12(1):1–16. 10.1186/1471-2458-12-679 [accessed 6 June 2020]. 10.1186/1471-2458-12-679 PMC3563464 22905965

[11] The Editorial Board of Wall Street Journal. Germany’s R0 coronavirus experiment. WSJ Opinion. April 28, 2020. https://www.wsj.com/articles/germanys-r0-coronavirus-experiment-11588115565 [accessed 2020 Jun 6].

[12] The Government of the UK. Guidance: The R number and growth rate in the UK. Last updated June 25, 2020. https://www.gov.uk/guidance/the-r-number-in-the-uk [accessed 3 July 2020].

[13] Grossman, M. On the concept of health capital and the demand for health. J Polit Econ 1972; 80(2): 223–225. https://www.journals.uchicago.edu/doi/pdfplus/10.1086/259880 [accessed 2 July 2020].

[14] Wibowo D, Tisdell C. Health, safe water and sanitation: a cross-sectional health production function for Central Java, Indonesia. Bull World Health Organ 1993; 71(2): 237–245. https://apps.who.int/iris/bitstream/handle/10665/47810/bulletin_1993_71%282%29_237-245.pdf?sequence=1&isAllowed=y [accessed 2 July 2020]. PMC2393453 8490988

[15] Kucharski AJ, Russel TW, Diamond C, et al. Early dynamics of transmission and control of COVID-19: a mathematical modelling study. Lancet Infect Dis 2020; 20(5):553–558. https://www.thelancet.com/article/S1473-3099(20)30144-4/fulltext [accessed 3 June 2020], doi: 10.1016/S1473-3099(20)30144-4. 10.1016/S1473-3099(20)30144-4 PMC7158569 32171059

[16] Shishkin D, Olifer, A. Point elasticity versus arc elasticity: on different approaches to teaching elasticity in principles courses. J Econ Econ Educ Res 2017; 18(2):1–7. https://www.abacademies.org/articles/Point-Elasticity-Versus-Arc-Elasticity-1533-3604-18-2-111.pdf [accessed 18 June 2020].

[17] WHO. WHO Coronavirus (COVID-19) Dashboard. https://covid19.who.int [accessed 19 March 2021].

[18] Wibowo, D.H. Data supporting the article: when can physical distancing be relaxed? A health production function approach for COVID-19 control policy. Figshare, 2021. 10.6084/m9.figshare.13047575. 10.1186/s12889-021-11088-x PMC8170438 34078329

