# Supplementary material for: When can physical distancing be relaxed? A health production function approach for COVID-19 control policy
Source: BMC Public Health. 2021 Jun 2;21:1037. doi: 10.1186/s12889-021-11088-x (PMC8170438; doi:10.1186/s12889-021-11088-x)
Supplement: Supplementary file 1 — Additional file 1. Cumulative number of COVID-19 cases as a short-run health production function; a formal mathematical presentation of the method used in this study. [file 12889_2021_11088_MOESM1_ESM.docx]

**Additional file 1: Cumulative number of COVID-19 cases as a short-run health production function**

Consider the Susceptible-Infected-Recovered (SIR) model expressed in the following deterministic, nonlinear system of ordinary differential equations:

$$\frac{dS}{dt}=- \beta S\left( t \right) I\left( t \right) (1)$$

$$\frac{dI}{dt}= \beta S\left( t \right) I\left( t \right)-\gamma I\left( t \right) (2)$$

$$\frac{dR}{dt}= \gamma I\left( t \right) (3)$$

where *S(t)* = the susceptible, *I(t)* = the infected, *R(t)* the recovered, *t* = time, $\beta$ = the transmission (infection) rate, $\gamma$ = the mean recovery rate; both $\beta$ and $\gamma$ are positive constants. The initial conditions are given by *S(0)* = *N_S(0)_*$\geq$0, *I(0)* = *N_I(0)_*$\geq$0, and *R(0)* = *N_R(0)_*$\geq$0, where *N_S(0)_*+ *N_I(0)_*+ *N_R(0)_*= *N* $\in\mathfrak{R}$.

Summing up (1)-(3) gives:

$$\frac{d}{dt} [S\left( t \right)+I\left( t \right)+R\left( t \right)]=0 (4)$$

And integrating (4) yields:

$$S\left( t \right)+I\left( t \right)+R\left( t \right)=N; \forall t\geq0 (5)$$

Now define *Y(t)* as a twice-differentiable function given by:

$$Y\left( t \right)=\int_{t=0}^{t=\tau} I\left( t \right) dt; I\left( t \right)\geq0 (6)$$

We can immediately see that for $\forall\tau>0$, *Y(*$\tau)$ is the cumulative number of the infected cases from *t*=0 to *t*=$\tau$. Employing *t* as the input variable, from the production theory viewpoint, *Y(t)* is actually the total product function of the infected at time *t*, $\forall I\left( t \right)\geq0.$ The marginal product of the infected (*MY*), the average product of the infected (*AY*) and the production elasticity of the infected with respect to (w.r.t) time *t* ($ℇ$*t*) are given as follows, respectively:

$$MY= \frac{dY}{dt}= I\left( t \right); AY= \frac{Y(t)}{t};\mathrm{and} ℇt=I(t) \frac{t}{Y(t)} (7)$$

with $ℇ$*t* defined as the percentage change in *Y(t)* for every 1% change in *t*. Thus $ℇ$*t* = $\frac{\%dY(t)}{\%dt}$ = $\frac{dY(t)/Y(t)}{dt/t}$ = $\frac{MY}{AY}$. From (7) it follows that $ℇ$*t* = 0 when *I(t)* = 0 and $ℇ$*t* = 1 when *Y(t)* = *I(t) t*.

The main departure of *Y(t)* from the standard short-run production function in economics is that *MY*= $\frac{dY}{dt}$ = *I(t)* $\geq0$. It means that as *t* increases, *Y(t)* exhibits no downward curve. This property of *Y(t)* represents the fact that the cumulative number of cases of a disease such as COVID-19 does not decrease, unless there is a change in the definition of confirmed cases or an incorrect recording of data. As *MY*= $\frac{dY}{dt}$ = *I(t)* $\geq0$, it follows from (7) that $ℇ$*t*$\geq0$.

Because the number of confirmed COVID-19 cases is reported daily, and recognizing that these data are the best available guesses of the true number of the infected, for practical policy purposes we can let *dt* = 1 reporting day. Without needing to estimate the functional form of *Y(t)* first, it is straightforward to see that:

$$MY=I\left( \tau\right)= Y\left( \tau\right)-Y\left( \tau-1 \right); AY=\frac{Y\left( \tau\right)}{\tau};\mathrm{and} ℇ\tau=\frac{I\left( \tau\right)\tau}{Y\left( \tau\right)} (8)$$

where *Y(*$\tau)$ = the reported number of cumulative COVID-19 cases at time $\tau$, *I(*$\tau)$ = the reported number of daily COVID-19 cases at time $\tau$, $\tau=$ the $\tau$-th day of the COVID-19 pandemic (as officially reported), *Y(0)* = *I(0)* = 0 and *Y(1)* = I(1).

In economics, $ℇ\tau$ as expressed by (8) is known as point elasticity at $\tau$. For the reason of rigor in definition, the concept of arc elasticity was introduced in economics. Measured by the midpoint method, the arc elasticity version of $ℇ\tau$ is given by:

$$ℇ\tau=\frac{Y\left( \tau\right)-Y\left( \tau-1 \right)}{I\left( \tau\right)- I\left( \tau-1 \right)} \frac{I\left( \tau\right)+I\left( \tau-1 \right)}{Y\left( \tau\right)+ Y\left( \tau-1 \right)} (9)$$

The arc elasticity (9) is known as the Allen’s formula of elasticity, and is considered the most consistent conception of elasticty^16^.

**Probability of the near term’s new COVID-19 cases given the production elasticity**

Let *I** be the maximum number of daily (new) COVID-19 cases at time *t* set as a policy target. *I** ideally represents the number of daily cases that a health system can cope with, but it could also be determined arbitrarily based on, say, a socio-political process.

Using Bayesian inferences, we can estimate the probability of the number of near term’s new COVID-19 cases does not exceed *I**, conditional on a given range of production elasticity at time $\tau$, denoted $ℇ\tau$range, as follows:

$$P\left( I\left( \tau+1 \right)\leq I^{*} \right| ℇ\tau range)=\frac{P\left( I\left( \tau+1 \right)\leq I^{*} \right) P\left( ℇ\tau range \right| I(\tau+1)\leq I^{*})}{P(ℇ\tau range)} (10)$$

A rational policy maker will consider relaxing physical distancing measures only when the probability of meeting the policy target *I** is high, *vice versa.*

More cautious policy makers might include additional policy parameters such as that “the number of daily new cases is constant or declining”. In this case, we need to modify formula (10) slightly to have:

$P\left( I\left( \tau+1 \right)\leq I^{*}, I\left( \tau+1 \right)\leq I(\tau) \right| ℇ\tau range)=\frac{P\left( I\left( \tau+1 \right)\leq I^{*}, I\left( \tau+1 \right)\leq I(\tau\right) P\left( ℇ\tau range \right| I\left( \tau+1 \right)\leq I^{*}, I\left( \tau+1 \right)\leq I(\tau))}{P(ℇ\tau range)} (11)$

**Exponential moving average (EMA)**

Because *Y(t)* and *I(t)* are time-series variables, one may smooth out the data by use of, for example, Exponential Moving Average (EMA) to have clearer data trends. For any time-series variable *X*, the *k*-day EMA of *X* is given by:

$EMAX\left( t \right)=\left( \omega\left( X\left( t \right)-EMAX\left( t-1 \right) \right) \right)+EMAX\left( t-1 \right);\omega=\frac{2}{k+1};t=k+1, k+2, \ldots, k+\tau(12)$

For *t=k+1*, the value of EMAX(*k*) is not defined. So, EMAX(*k*) is given by $\frac{1}{k}\sum_{t=1}^{k} X(t)$.
