# Supplementary material for: When can physical distancing be relaxed? A health production function approach for COVID-19 control policy
Source: BMC Public Health. 2021 Jun 2;21:1037. doi: 10.1186/s12889-021-11088-x (PMC8170438; doi:10.1186/s12889-021-11088-x)

**The initial period’s results**

To test this approach, the author initially analyses COVID-19 cases in France, Germany, Italy, the UK and the US. The method is then applied to Indonesia as a developing country example, given the author’s familiarity with its health data collection system. The first manuscript was completed in July 2020, covering a period from the first day a confirmed case is recorded until June 30, 2020, termed henceforth the “initial period”. All data are obtained from the WHO Coronavirus (COVID-19) Dashboard.

**The state of COVID-19 transmission**

Descriptive statistics of the data are given in Table 1. Figure 1 presents the 5-day EMA curves of *Y(t)*, *MY*=*I(t)*, and *AY*.

*(Table 1 and Figure 1 can be placed here)*

From France’s, Germany’s, Italy’s and the UK’s curves in Figure 1, it is obvious that *Y(t)*, *MY*, and *AY* have the curvature of short-run total, marginal, and average products, respectively. With regard to the state of transmission, these countries have all reached *t_1_* with Italy being the earliest one and the UK the latest one.

The US on the contrary has not reached *t_1_*. It appeared to reach *t_1_* on April 27, 2020 and the number of daily cases was on the decline until around the first week of June 2020. But this decline was not sustained, the US’ *I(t)* was rising to a new not-yet-known height in June 2020. On the other hand, the US’ AY continued to rise during the period of analysis. All these explain why *Y(t)* in the US had yet to exhibit the standard total product curve.

With regard to t_2_, France, Germany, and Italy reached it in the end of April 2020, while the UK in the final week of May 2020. On May 7, 29 and 31 France recorded large jumps in its daily cases, resulting in *MY*>*AY*. But France’s *AY* has been declining from April 26-28, and its *MY* is lower than its *AY* from May 7 to June 30, except on the days where the jumps are recorded. So the jumps are seen as outliers.

With regard to t_3_, none of the countries studied has suppressed their *I(t)* to zero. So, none of them has arrived at the steady-state of *Y(t)*.

**Production elasticities**

Table 2 presents the arc production elasticity ($ℇ$*t*) for these countries. Point elasticity values are also presented for comparative purpose. In general, arc elasticities are larger than point elasticities for all countries. Italy has the smallest $ℇ$*t*, with a mean of 2.92. This means that for every one per cent change in time, Italy has 2.92 per cent additional COVID-19 cases. The US exhibits the largest $ℇ$*t* with a maximum value of 36.11.

*(Table 2 can be placed here)*

The UK’s $ℇ$*t* has a mean of 3.62, larger than France’s, Germany’s and Italy’s. With a lower coefficient of variation (CV), the UK’s $ℇ$*t* is less dispersed around its mean value. These results reflect the UK’s persistently higher daily COVID-19 cases, and its inferior ability to suppress both *I(t)* and $ℇ$*t* compared to France, Germany and Italy.

**Probability of a policy target**

The next analysis is not relevant for the US because it has yet to reach *t_1_*_._  Now let’s assume that based on the latest *I(t)* records, France, Germany and Italy set the policy target *I** arbitrarily at 500 daily-cases. Table 3 presents the probability of *I(t+1)*$\leq$*I**, given a range of $ℇ$*t*.

*(Table 3 can be placed here)*

For $ℇ$*t*>1 (the “yellow zone”), the probability of *I(t+1)*$\leq$500 is zero for these countries. This means, even though France, Germany, and Italy have reached *t_1_*, they have a zero chance of supressing their near term’s daily-cases below or equal to 500. After reaching *t_1_*, with $ℇ$*t*>1 they only have a probability of greater than zero if *I** is set higher than 500. For example, France will have a probability of 0.03 if *I** is set at 900. This explains why relaxation is not recommended in this transmission state.

If a country reaches t_2_, they have $0\leqℇ$*t*$\leq$1. As shown in Table 3, the probabilities of *I(t+1)*$\leq$500 are 0.69, 0.41, and 0.53 for France, Germany and Italy, respectively. If policy makers aim at having a larger probability, they need to set a lower elasticity range. Table 3 presents the probabilities if the range is set at $0\leqℇ$*t*$\leq$0.5 and $0\leqℇ$*t*$\leq$0.3. For $0\leqℇ$*t*$\leq$0.3, France has a probability of 0.95 (20 out of 21 cases), Germany 0.94 (17 out of 18 cases) and Italy 0.91 (32 out of 35 cases).

If *I(t+1)*$\leq$*I(t)* is put as an additional target, the probabilities return lower values, except for $ℇ$*t*>1 where the probabilities are again zero. For $0\leqℇ$*t*$\leq$0.3, Germany and Italy have a probability of 0.56 (10 out of 18 cases) and 0.63 (22 out of 35 cases), respectively. Against expectation, France’s probability of 0.33 (seven out of 21 cases) is lower than its probability in the $0\leqℇ$*t*$\leq$0.5 range. France’s erratic data on May 6-7, 29, 31, June 3 (a negative entry for *I(t)*), and June 22, 26, 28, 29 cause this irregularity, and an EMA longer than 5 days is needed to smooth out the data.

The UK shows similar results but with a much higher *I** of 1,000. This is because for *I**< 1,000 the UK has no or only few records that meet the threshold. For example, the UK has no records that meet *I**=500 because this level is unattainable. For *I**=1,000 the UK has 12 data records, returning a probability of 0.82 at $0\leqℇ$*t*$\leq$0.5. For the *I(t+1)*$\leq$*I** and *I(t+1)*$\leq$*I(t)* policy target, at $0\leqℇ$*t*$\leq$0.5 the UK’s probability is 0.55 (six out of 11 cases). The $0\leqℇ$*t*$\leq$0.3 range is not applicable for the UK because its lowest elasticity for the April 25-June 30, 2020 period was 0.33.

**Application to a developing country: Indonesia**

In January and February 2020 Indonesia denied that the country has a COVID-19 case. When the central government finally announced the “first” COVID-19 case on March 2, opportunity to estimate *R_0_* more accurately has been wasted. Consequently, Indonesia has no reliable estimates of *R* to assess its state of transmission.

On the other hand, the results from France, Germany, Italy and the UK show that short-run health production function and elasticity can be used to assess the state of transmission. As shown by Table 1 and Figure 1, Indonesia has not reached *t_1_*. It means, the country is still in a transmission state where physical distancing needs to be applied to bring down the number of daily-cases. Yet on June 1, 2020 Indonesia began to relax physical distancing in some of its provinces in order to “save” the economy. Unsurprisingly, Indonesia’s daily-cases keep rising after the relaxation.

**Discussion**

This study demonstrates how short-run health production function is employed to assess the state of COVID-19 transmission, using only data on the cumulative number of cases and the recording dates. The data are processed in a relatively simple way in Microsoft Excel. To view how the calculations are done, see Additional files 3-8. This simple approach can be performed at minimal costs in developing countries. Needless to say that the accuracy of the results depends on data quality.

This study also shows that relaxing physical distancing measures can only be considered when the state of transmission is in the “green zone”. In this zone the probability of maintaining a relatively low number of near term’s daily COVID-19 cases, at a given elasticity range, is relatively high. In the “yellow zone” the probability is zero or near zero.

As of June 30, 2020, France, Germany, Italy, and the UK have all arrived at the “green zone”. With a policy target of 500 daily-cases, France, Germany, and Italy need to have an elasticity of ≤ 0.5 to return a probability larger than 0.7. At a higher elasticity, their probability can fall below 0.5. If the policy target includes “keeping a constant or declining number of daily-cases”, their probabilities are below 0.5, unless the elasticity is kept at ≤ 0.3. In other words, France, Germany and Italy still have a high risk of their daily-cases rising.

The UK must make do at a higher target of 1,000 daily-cases, and also has a high risk of its daily-cases rising. The US and Indonesia are still in the “red zone”, hence, physical distancing measures need to be applied in these countries

|  | **France** | **Germany** | **Italy** | **The UK** | **The US** | **Indonesia** |
| --- | --- | --- | --- | --- | --- | --- |
| Recording dates | Jan 24-  June 30, 2020 | Jan 28-  June 30. 2020 | Jan 29-  June 30, 2020 | Feb 1-  June 30, 2020 | Jan 20-  June 30, 2020 | March 2-  June 30, 2020 |
|  |  |  |  |  |  |  |
|  |  |  |  |  |  |  |
| Number of recording days (t) | 159 | 155 | 154 | 151 | 163 | 121 |
|  |  |  |  |  |  |  |
| Cumulative number of cases, Y(t), on June 30, 2020 |  |  |  |  |  |  |
| Orignal data | 156887 | 194259 | 240436 | 283545 | 2537636 | 55092 |
| EMA | 156177 | 193382 | 240052 | 282290 | 2456444 | 52750 |
|  |  |  |  |  |  |  |
| Number of daily-cases, MY=I(t), 5-day EMA |  |  |  |  |  |  |
| Mean | 1008 | 1281 | 1600 | 1920 | 15449 | 451 |
| Standard deviation | 1272 | 1603 | 1758 | 1744 | 12774 | 353 |
| Coefficient of variation | 126% | 125% | 110% | 91% | 83% | 78% |
| Maximum value | 4927 | 5828 | 5774 | 5007 | Not applicable | Not  Applicable |
| Date of maximum value (*t_1_*) | April 1 | April 5 | March 29 | April 25 | Not applicable | Not  Applicable |
|  |  |  |  |  |  |  |
| Average product of the infected, AY, 5-day EMA |  |  |  |  |  |  |
| Mean | 694 | 935 | 1284 | 1107 | 6067 | 177 |
| Standard deviation | 543 | 699 | 863 | 906 | 5855 | 131 |
| Coefficient of variation | 78% | 75% | 67% | 82% | 97% | 74% |
| Maximum value | 1297 | 1705 | 2189 | 2127 | Not applicable | Not  applicable |
| Date of  maximum value (*t_2_*) | April 26-28 | April 26-27 | April 29-30 | May 24-25 | Not applicable | Not  applicable |

**Tables and figures**

Table 1. **Descriptive statistics (… - June 30, 2020)**

Table 2. **The elasticity of production (… - June 30, 2020)**

|  | **France** | **Germany** | **Italy** | **The UK** | **The US** | **Indonesia** |
| --- | --- | --- | --- | --- | --- | --- |
| Arc elasticity of production, 5-day EMA |  |  |  |  |  |  |
| Mean | 3.36 | 3.18 | 2.92 | 3.62 | 4.97 | 2.80 |
| Standard deviation | 4.57 | 4.45 | 4.59 | 3.65 | 6.02 | 0.82 |
| Coefficient of variation | 136% | 140% | 157% | 101% | 121% | 29% |
| Maximum value | 18.52 | 18.41 | 31.01 | 13.75 | 36.11 | 5.16 |
| Minimum value *) | 0.04 | 0.01 | 0.00 | 0.05 | 0.10 | 0.44 |
|  |  |  |  |  |  |  |
| Point elasticity of production, 5-day EMA |  |  |  |  |  |  |
| Mean | 3.00 | 2.82 | 2.52 | 3.26 | 4.45 | 2.58 |
| Standard deviation | 3.94 | 3.82 | 3.51 | 3.19 | 4.99 | 0.69 |
| Coefficient of variation | 131% | 135% | 139% | 98% | 112% | 27% |
| Maximum value | 14.71 | 15.49 | 19.40 | 11.69 | 27.59 | 4.35 |
| Minimum value *) | 0.04 | 0.01 | 0.00 | 0.05 | 0.10 | 0.46 |

Note: *) It excludes minimum values in the beginning of transmission.

Table 3. **Probability of a policy target**

|  | **France** | **Germany** | **Italy** | **The UK** | **The US** | **Indonesia** |
| --- | --- | --- | --- | --- | --- | --- |
| Policy (daily-cases) target, I* | 500 | 500 | 500 | 1000 | This analysis is not applicable for the US | This analysis is not applicable for Indonesia |
|  |  |  |  |  |  |  |
| Probability of I (t+1) ≤ I*, if: | **3** |  |  |  |  |  |
| ɛt > 1.0 | 0.00 | 0.00 | 0.00 | 0.00 |  |  |
| 0 ≤ ɛt ≤ 1.0 | 0.69 | 0.41 | 0.53 | 0.34 |  |  |
| 0 ≤ ɛt ≤ 0.5 | 0.89 | 0.58 | 0.70 | 0.82 |  |  |
| 0 ≤ ɛt ≤ 0.3 | 0.95 | 0.94 | 0.79 | Not applicable |  |  |
|  |  |  |  |  |  |  |
|  |  |  |  |  |  |  |
| Probability of I (t+1) ≤ I* and  I (t+1) ≤ I(t), if: |  |  |  |  |  |  |
| ɛt > 1.0 | 0.00 | 0.00 | 0.00 | 0.00 |  |  |
| 0 ≤ ɛt ≤ 1.0 | 0.38 | 0.29 | 0.37 | 0.26 |  |  |
| 0 ≤ ɛt ≤ 0.5 | 0.48 | 0.40 | 0.48 | 0.55 |  |  |
| 0 ≤ ɛt ≤ 0.3 | 0.33 | 0.56 | 0.63 | Not applicable |  |  |
|  |  |  |  |  |  |  |

**Figure 1: Cumulative number of cases, daily-cases, and average product of the infected (… - June 2020)**


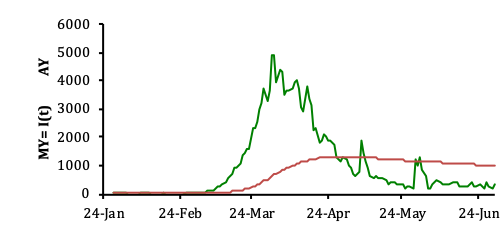

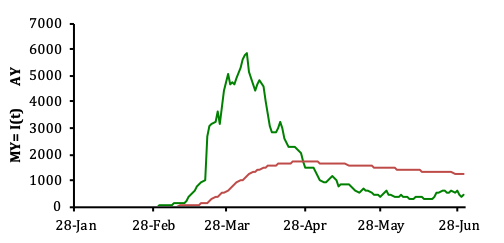

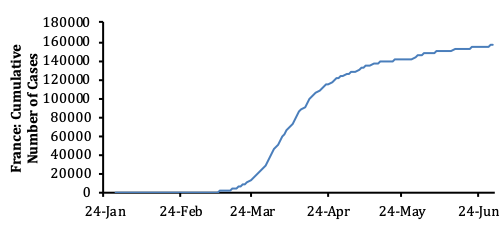


t1

t2


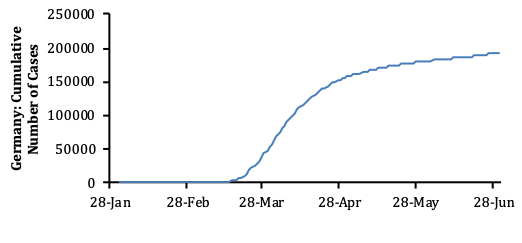


t1

t2


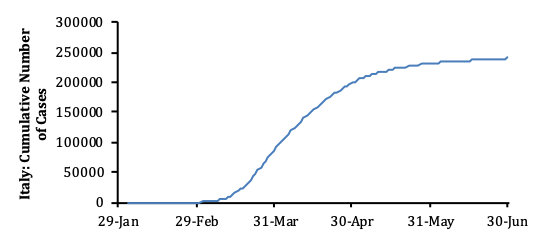


t1

t2


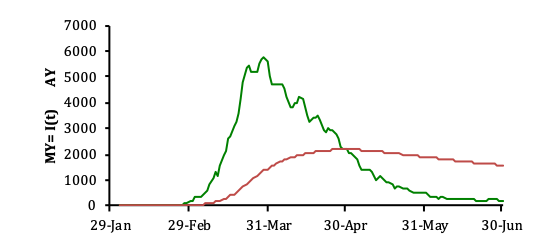

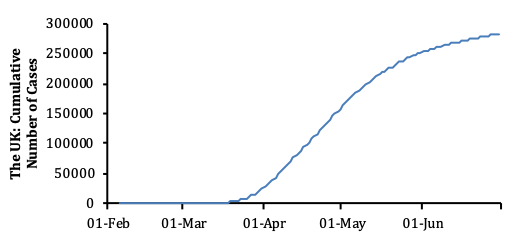


t1

t2


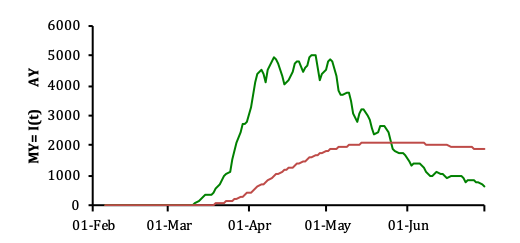

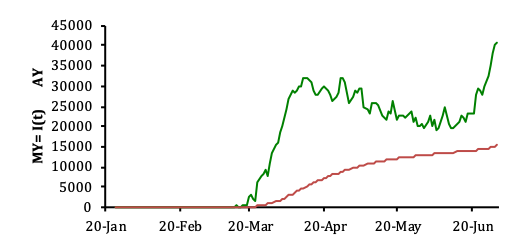

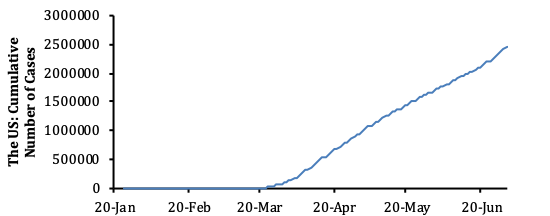

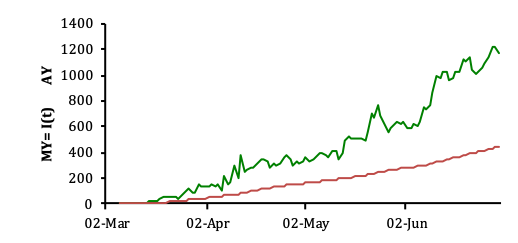

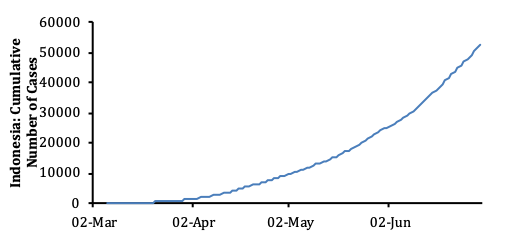

Supplement: Supplementary file 2 — Additional file 2. Figures; The initial period’s results. [file 12889_2021_11088_MOESM2_ESM.docx]
