# Supplementary material for: When can physical distancing be relaxed? A health production function approach for COVID-19 control policy
Source: BMC Public Health. 2021 Jun 2;21:1037. doi: 10.1186/s12889-021-11088-x (PMC8170438; doi:10.1186/s12889-021-11088-x)
Supplement: Supplementary file 10 — Additional file 10. Eviews and matlab exercises; Eviews and matlab results for the UK. [file 12889_2021_11088_MOESM10_ESM.docx]

**Eviews and Matlab exercises for the UK data**

The author’s intention is to show how functions and parameters obtained from simple methods making use of short-run health production function can be utilised to assess the state of COVID-19 transmission and to determine risk-based physical distancing relaxation policy. Derived with the aid of a simple “bridge” between epidemiological models and production economics, the methods require only official daily data of COVID-19 cases. The methods, in the author’s view, is useful for developing countries which for various reasons are unable to estimate the reproduction number *R* reliably. Simplicity and minimal data requirement are key features of the methods.

More advanced statistical techniques can certainly be employed to yield the best-fitted functional form with unbiased regression estimators. But doing so requires more efforts from health officials which include time, resources, skills, and experience. To show this, the author performs regression and curve-fitting exercises to the UK data using Eviews and Matlab, respectively. Given the curvature of the UK’s cumulative number of COVID-19 cases from February 1, 2020 to February 28, 2021, the author examines second to sixth order polynomial functions.

The regressions return a very high value of adjusted R-square, ranging from 0.9493 to 0.9969, suggesting that almost all variation of *Y(t)* can be explained by *t* and its polynomials, and hence the functions appear to be very well-fitted. The prob(F-statistic) returns a zero value for all polynomial specifications, meaning that all the *t* variables included significantly affect *Y(t)* at 0.00 significance level.

But the specifications have a major statistical flaw. They all suffer from a severe positive autocorrelation as indicated by their Durbin-Watson statistic being close to zero. This problem makes the ordinary least squares (OLS) inefficent and not the best linear unbiased estimators (BLUE), with invalid standard errors and t-statistics. In addition to this, the regressions also return very large positive or negative intercepts, which are unrealistic.

Because at *t* = 0, *Y(t) =* 0, it is logical to set the intercepts at zero. While statistically such an exercise is not encouraged, the author runs a regression through the origin (RTO) for curiosity and analytical completeness. The regressions return similar results to the ones with an intercept, with a very high adjusted R-square of 0.9315 to 0.9968 and a severe positive autocorrelation.

A power function is also regressed. It returns a lower adjusted R-square of 0.9038 and also suffers from a severe positive autocorrelation. Note that power functions give a constant production elasticity, making it unattractive for assessing COVID-19’s state of transmission.

Because this study collapses all health inputs into the time variable, the autocorrelation might result from the problem of omitted independent variables. Thus, one solution to this is to employ a long run (multiple inputs) health production function. Another solution is to run specifications such as autoregressive time series model of order *p* or AR(*p*), autoregressive moving average or ARMA(*p,q*), and autoregressive integrated moving average or ARIMA(*p,d,q*). But doing these remedies changes the focus of the study significantly and eliminates the simplicity feature of the methods. For this reason, the author reserves these remedies for future research.

The author also runs a curve-fitting exercise in Matlab. Because Matlab does not automatically show the residual sum of squares (RSS), we need to review the plots visually. From this visual review, it seems that the best-fitted function takes the form of *Y(t) = -0.000000035t^6^ + 0.000033t^5^ – 0.0098t^4^ + 1.09t^3^ – 21.21t^2^ + 16.59t – 4429.56.* A manual check of the function, however, shows that the function very poorly estimates the original data. It yields negative entries of – 4391 to – 368 between February 1-11, 2020. Note that *Y(t)* shall not take negative values. It also gives too large numbers for the later dates of the analysis. For example, for February 28, 2021, it yields a very inaccurate *Y(t)* of over 147 million cases with over 2 million daily cases. See Additional file 6, worksheet “Eviews-Matlab estimators”.

These exercises clearly show the large efforts needed by developing country officials to run more advanced statistics. To keep the methods simple, the author intentionally employs minimal statistics. Detailed Eviews and Matlab results are presented on pages 2-9 of this Additional file, while the Matlab code is on page 10.

**Eviews results**

**The UK polynomial regression (Feb 1, 2020 – Feb 28, 2021)**

**2^ND^ Order**

Dependent Variable: Y

Method: Least Squares

Date: 03/28/21 Time: 06:17

Sample: 2/01/2020 2/28/2021

Included observations: 394

Variable Coefficient Std. Error t-Statistic Prob.

T -11368.32 499.9146 -22.74053 0.0000

T2 52.27734 1.225613 42.65403 0.0000

C 502836.1 42759.44 11.75965 0.0000

R-squared 0.949559 Mean dependent var 973007.9

Adjusted R-squared 0.949301 S.D. dependent var 1250123.

S.E. of regression 281482.0 Akaike info criterion 27.94111

Sum squared resid 3.10E+13 Schwarz criterion 27.97139

Log likelihood -5501.399 Hannan-Quinn criter. 27.95311

F-statistic 3680.348 Durbin-Watson stat 0.001352

Prob(F-statistic) 0.000000

**3^rd^ Order**

Dependent Variable: Y

Method: Least Squares

Date: 03/28/21 Time: 06:16

Sample: 2/01/2020 2/28/2021

Included observations: 394

Variable Coefficient Std. Error t-Statistic Prob.

T 8501.728 610.6198 13.92311 0.0000

T2 -73.32322 3.589763 -20.42564 0.0000

T3 0.211984 0.005974 35.48233 0.0000

C -155361.1 27886.10 -5.571274 0.0000

R-squared 0.988070 Mean dependent var 973007.9

Adjusted R-squared 0.987979 S.D. dependent var 1250123.

S.E. of regression 137065.8 Akaike info criterion 26.50441

Sum squared resid 7.33E+12 Schwarz criterion 26.54478

Log likelihood -5217.369 Hannan-Quinn criter. 26.52041

F-statistic 10767.28 Durbin-Watson stat 0.006340

Prob(F-statistic) 0.000000

**4^th^ Order**

Dependent Variable: Y

Method: Least Squares

Date: 03/28/21 Time: 06:16

Sample: 2/01/2020 2/28/2021

Included observations: 394

Variable Coefficient Std. Error t-Statistic Prob.

T 11901.28 1211.924 9.820154 0.0000

T2 -111.9378 12.45452 -8.987726 0.0000

T3 0.363924 0.047346 7.686505 0.0000

T4 -0.000192 5.95E-05 -3.234390 0.0013

C -223267.8 34641.23 -6.445145 0.0000

R-squared 0.988383 Mean dependent var 973007.9

Adjusted R-squared 0.988263 S.D. dependent var 1250123.

S.E. of regression 135432.9 Akaike info criterion 26.48295

Sum squared resid 7.14E+12 Schwarz criterion 26.53341

Log likelihood -5212.141 Hannan-Quinn criter. 26.50294

F-statistic 8273.987 Durbin-Watson stat 0.006138

Prob(F-statistic) 0.000000

**5^th^ Order**

Dependent Variable: Y

Method: Least Squares

Date: 03/28/21 Time: 06:15

Sample: 2/01/2020 2/28/2021

Included observations: 394

Variable Coefficient Std. Error t-Statistic Prob.

T -15841.73 1275.685 -12.41822 0.0000

T2 377.2202 19.94971 18.90856 0.0000

T3 -2.932107 0.127872 -22.92995 0.0000

T4 0.009189 0.000357 25.76169 0.0000

T5 -9.50E-06 3.59E-07 -26.43269 0.0000

C 148499.3 25047.61 5.928680 0.0000

R-squared 0.995852 Mean dependent var 973007.9

Adjusted R-squared 0.995799 S.D. dependent var 1250123.

S.E. of regression 81030.17 Akaike info criterion 25.45814

Sum squared resid 2.55E+12 Schwarz criterion 25.51870

Log likelihood -5009.254 Hannan-Quinn criter. 25.48214

F-statistic 18630.71 Durbin-Watson stat 0.008430

Prob(F-statistic) 0.000000

**6^th^ Order**

Dependent Variable: Y

Method: Least Squares

Date: 03/28/21 Time: 06:15

Sample: 2/01/2020 2/28/2021

Included observations: 394

Variable Coefficient Std. Error t-Statistic Prob.

T 16.58603 1781.016 0.009313 0.9926

T2 -21.20615 39.10444 -0.542295 0.5879

T3 1.089733 0.370969 2.937535 0.0035

T4 -0.009875 0.001706 -5.787285 0.0000

T5 3.30E-05 3.75E-06 8.787762 0.0000

T6 -3.58E-08 3.15E-09 -11.36076 0.0000

C -4429.560 25551.79 -0.173356 0.8625

R-squared 0.996889 Mean dependent var 973007.9

Adjusted R-squared 0.996841 S.D. dependent var 1250123.

S.E. of regression 70260.23 Akaike info criterion 25.17541

Sum squared resid 1.91E+12 Schwarz criterion 25.24605

Log likelihood -4952.555 Hannan-Quinn criter. 25.20340

F-statistic 20671.62 Durbin-Watson stat 0.008109

Prob(F-statistic) 0.000000

**The UK polynomial regression through the origin (RTO) (Feb 1, 2020 – Feb 28, 2021)**

**2^nd^ Order**

Dependent Variable: Y

Method: Least Squares

Date: 03/28/21 Time: 19:05

Sample: 2/01/2020 2/28/2021

Included observations: 394

Variable Coefficient Std. Error t-Statistic Prob.

T -6269.871 289.2054 -21.67965 0.0000

T2 41.50748 0.946421 43.85729 0.0000

R-squared 0.931720 Mean dependent var 973007.9

Adjusted R-squared 0.931545 S.D. dependent var 1250123.

S.E. of regression 327080.1 Akaike info criterion 28.23886

Sum squared resid 4.19E+13 Schwarz criterion 28.25905

Log likelihood -5561.056 Hannan-Quinn criter. 28.24686

Durbin-Watson stat 0.000947

**3^rd^ Order**

Dependent Variable: Y

Method: Least Squares

Date: 03/28/21 Time: 19:04

Sample: 2/01/2020 2/28/2021

Included observations: 394

Variable Coefficient Std. Error t-Statistic Prob.

T 5548.136 314.4103 17.64617 0.0000

T2 -58.34943 2.469379 -23.62919 0.0000

T3 0.189843 0.004629 41.01167 0.0000

R-squared 0.987121 Mean dependent var 973007.9

Adjusted R-squared 0.987055 S.D. dependent var 1250123.

S.E. of regression 142233.6 Akaike info criterion 26.57591

Sum squared resid 7.91E+12 Schwarz criterion 26.60619

Log likelihood -5232.455 Hannan-Quinn criter. 26.58791

Durbin-Watson stat 0.005586

**4^th^ Order**

Dependent Variable: Y

Method: Least Squares

Date: 03/28/21 Time: 19:00

Sample: 2/01/2020 2/28/2021

Included observations: 394

Variable Coefficient Std. Error t-Statistic Prob.

T 5110.140 629.1097 8.122812 0.0000

T2 -51.68782 8.646843 -5.977652 0.0000

T3 0.160292 0.037049 4.326438 0.0000

T4 4.00E-05 4.97E-05 0.803920 0.4219

R-squared 0.987142 Mean dependent var 973007.9

Adjusted R-squared 0.987043 S.D. dependent var 1250123.

S.E. of regression 142297.9 Akaike info criterion 26.57933

Sum squared resid 7.90E+12 Schwarz criterion 26.61970

Log likelihood -5232.129 Hannan-Quinn criter. 26.59533

Durbin-Watson stat 0.005706

**5^th^ Order**

Dependent Variable: Y

Method: Least Squares

Date: 03/28/21 Time: 18:59

Sample: 2/01/2020 2/28/2021

Included observations: 394

Variable Coefficient Std. Error t-Statistic Prob.

T -9254.935 653.8655 -14.15419 0.0000

T2 288.1752 13.69471 21.04281 0.0000

T3 -2.424254 0.099020 -24.48251 0.0000

T4 0.007902 0.000295 26.77181 0.0000

T5 -8.30E-06 3.10E-07 -26.77064 0.0000

R-squared 0.995476 Mean dependent var 973007.9

Adjusted R-squared 0.995430 S.D. dependent var 1250123.

S.E. of regression 84512.07 Akaike info criterion 25.53979

Sum squared resid 2.78E+12 Schwarz criterion 25.59025

Log likelihood -5026.338 Hannan-Quinn criter. 25.55978

Durbin-Watson stat 0.007671

**6^th^ Order**

Dependent Variable: Y

Method: Least Squares

Date: 03/28/21 Time: 18:58

Sample: 2/01/2020 2/28/2021

Included observations: 394

Variable Coefficient Std. Error t-Statistic Prob.

T -252.8431 868.6809 -0.291066 0.7712

T2 -16.08434 25.58529 -0.628656 0.5299

T3 1.046458 0.274068 3.818237 0.0002

T4 -0.009694 0.001348 -7.191738 0.0000

T5 3.26E-05 3.09E-06 10.54227 0.0000

T6 -3.55E-08 2.68E-09 -13.27510 0.0000

R-squared 0.996889 Mean dependent var 973007.9

Adjusted R-squared 0.996849 S.D. dependent var 1250123.

S.E. of regression 70172.36 Akaike info criterion 25.17041

Sum squared resid 1.91E+12 Schwarz criterion 25.23096

Log likelihood -4952.570 Hannan-Quinn criter. 25.19440

Durbin-Watson stat 0.008113

**The UK power function regression (Feb 1, 2020 – Feb 28, 2021)**

Dependent Variable: LNY

Method: Least Squares

Date: 03/29/21 Time: 08:38

Sample: 2/01/2020 2/28/2021

Included observations: 394

Variable Coefficient Std. Error t-Statistic Prob.

LNT 3.381362 0.055637 60.77543 0.0000

C -4.840821 0.282624 -17.12810 0.0000

R-squared 0.904054 Mean dependent var 12.01956

Adjusted R-squared 0.903810 S.D. dependent var 3.454956

S.E. of regression 1.071539 Akaike info criterion 2.981133

Sum squared resid 450.0931 Schwarz criterion 3.001317

Log likelihood -585.2832 Hannan-Quinn criter. 2.989131

F-statistic 3693.652 Durbin-Watson stat 0.024307

Prob(F-statistic) 0.000000

**Matlab curve-fitting and functional forms**

**The UK (Feb 1, 2020 – Feb 28, 2021)**

**Function: 52.28X^2^ – 11368.32X + 502836.07 Function: 0.21X^3^ – 73.32X^2^ + 8501.73X – 155361.09**

**Function:** **-0.000009X^5^ + 0.009X^4^ – 2.93X^3^ + 377.22X^2^ – 15841.73X + 148499.27**

**Function:** **-0.0002X^4^ + 0.36X^3^ – 111.94X^2^ + 11901.27X – 223267.78**

**Function: -0.000000035X^6^ + 0.000033X^5^ – 0.0098X^4^ + 1.09X^3^ – 21.21X^2^ + 16.59X – 4429.56**

**Matlab General Script Code:**

clc

clear all

load [(country name).mat]

Days= [(file name).NoOfDays]

Cases= [(file name).CumulativeCases]

plot(Days,Cases,'-','MarkerFaceColor','b')

xlabel('Number of Days (range of date)')

ylabel('Number of Cases (units)')

p= polyfit[Days,Cases,(select order number)];

pp= polyval(p,Days);

hold on

plot(Days,pp, '-')

legend('Number of Cases (actual)','[selected degree of polynomial}','Location','NorthWest')
